# Sensitivity of unconstrained quantitative magnetization transfer MRI to amyloid burden in preclinical Alzheimer’s disease

**DOI:** 10.1162/imag_a_00367

**Published:** 2024-11-25

**Authors:** Andrew Mao, Sebastian Flassbeck, Elisa Marchetto, Arjun V. Masurkar, Henry Rusinek, Jakob Assländer

**Affiliations:** Bernard and Irene Schwartz Center for Biomedical Imaging, Department of Radiology, New York University Grossman School of Medicine, New York, NY, United States; Center for Advanced Imaging Innovation and Research (CAI ^2^ R), Department of Radiology, New York University Grossman School of Medicine, New York, NY, United States; Vilcek Institute of Graduate Biomedical Sciences, New York University Grossman School of Medicine, New York, NY, United States; Alzheimer’s Disease Research Center, Center for Cognitive Neurology, New York University Grossman School of Medicine, New York, NY, United States; Department of Neurology, New York University Grossman School of Medicine, New York, NY, United States; Department of Neuroscience and Physiology, New York University Grossman School of Medicine, New York, NY, United States; Department of Psychiatry, New York University Grossman School of Medicine, New York, NY, United States

**Keywords:** Alzheimer’s disease, amyloid-beta, quantitative magnetization transfer, florbetaben, positron emission tomography

## Abstract

Magnetization transfer MRI is sensitive to semisolid macromolecules, includingamyloid beta, and has previously been used to discriminate Alzheimer’sdisease (AD) patients from controls. Here, we fit an unconstrained 2-poolquantitative MT (qMT) model, that is, without constraints on the longitudinalrelaxation rate$R_{1}^{\text{s}}$of semisolids, and investigate the sensitivity of the estimated parameters toamyloid accumulation in preclinical participants. We scanned 15 cognitivelynormal volunteers, of which 9 were amyloid positive by[^18^F]florbetaben PET. A 12 min hybrid-state qMT scan with aneffective resolution of 1.24 mm isotropic and whole-brain coverage was acquiredto estimate the unconstrained 2-pool qMT parameters. Group comparisons andcorrelations with florbetaben PET standardized uptake value ratios were analyzedat the lobar level. We find that the exchange rate and semisolid pool’s$R_{1}^{\text{s}}$were sensitive to the amyloid concentration, while morphometric measures ofcortical thickness derived from structural MRI were not. Changes in the exchangerate are consistent with previous reports in clinical AD, while changes in$R_{1}^{\text{s}}$have not been reported previously as its value is typically constrained in theliterature. Our results demonstrate that qMT MRI may be a promising surrogatemarker of amyloid beta without the need for contrast agents or radiotracers.

## 1 Introduction

Conformational abnormalities in the amyloid β (Aβ) protein are one ofthe defining pathological hallmarks of Alzheimer’s disease (AD) (Thal et al., 2002), along with tauabnormalities and neurodegeneration (Jack et al.,2018). In the extracellular space, 40–42 amino acid Aβpeptide fragments cleaved from amyloid precursor protein, known asAβ_40_and Aβ_42_, form soluble aggregates knownas oligomers, which further aggregate into insoluble fibrils and ultimately plaques(Karran et al., 2011). It is believedthat several species of Aβ aggregates have neurotoxic or inflammatory effects(Heppner et al., 2015) that ultimatelylead to tau hyperphosphorylation (Oddo et al.,2006), neurodegeneration, and cognitive symptoms (Bennett et al., 2004), though the exact mechanisms areunclear. Aβ accumulation begins in the neocortex (Fantoni et al., 2020), even in clinically asymptomaticparticipants, and can occur decades prior to the possible symptomatic onset ofAlzheimer’s dementia (Jack et al.,2010). Identifying individuals with a substantial cortical amyloid loadis necessary for many clinical reasons, such as the specific diagnosis of AD versusother dementias (Jack et al., 2018), asAβ deposits may be found incidentally in other forms of dementia but are nottheir primary pathological feature. Additionally, quantification of amyloid burdenenables the study of the impact of new disease-modifying therapies—such asemerging antiamyloid immunotherapeutics—which are being used to treat earlystages of AD and increasingly being studied in preclinical patient populations(Yadollahikhales & Rojas,2023).

The gold standard for noninvasive*in vivo*amyloid assessment ispositron emission tomography (PET) (Chapleau et al.,2022;Johnson et al., 2013;Rabinovici et al., 2023). The second generationof amyloid PET tracers includes [^18^F]florbetaben, which has beendemonstrated to have high sensitivity for fibrillar amyloid (Syed & Deeks, 2015). While cerebrospinal fluid(CSF) Aβ biomarkers are similarly sensitive and lumbar punctures enablesimultaneous access to tau biomarkers without requiring an additional procedure(Huszár et al., 2024;Palmqvist et al., 2015), intravenousradiotracer injection followed by PET imaging is less invasive. Additionally, PEToffers spatial localization of the Aβ signal which may confer the ability todetect regional amyloid depositions before pathological changes occur in the globalneocortical signal. However, PET has several drawbacks, including cost, the need forspecialized equipment, availability, limited spatial resolution, off-target binding,and ionizing radiation exposure.

An alternative, magnetic resonance imaging (MRI)-based technique that may be directlysensitive to the accumulation of extracellular protein deposits, such as Aβaggregates, is known as quantitative magnetization transfer (qMT) (Henkelman et al., 1993). MT methods sensitize the MRIsignal to a “semisolid” spin pool consisting of protons bound in largemacromolecules such as lipids (e.g., myelin) and proteins (e.g., bothAβ_40_and Aβ_42_aggregates), which exchangewith protons bound in the usual “free water” pool. MT’ssensitivity to the accumulation of insoluble Aβ plaques (note that solubleAβ species contribute minimally to the MT effect) was previously demonstratedin transgenic mice (Bigot et al., 2014;Pérez-Torres et al., 2014;Praet et al., 2016), and*invivo*studies using*quantitative*MT—whichdisentangles the biophysical contributions to the MT contrast—suggest thatthe “forward magnetization exchange rate” is the most discriminatoryqMT biomarker for classifying AD versus controls and the conversion from amnesticmild cognitive impairment (MCI) to AD (Duan et al.,2022;Giulietti et al., 2012;Makovac et al., 2018).

It is unclear from prior qMT studies whether the observed differences in the“forward exchange rate” arise from changes in the exchange rate (e.g.,due to the insolubility of Aβ plaques) or the macromolecular pool size (e.g.,from neurodegeneration). Additionally, prior studies usually constrain the value ofthe difficult-to-estimate semisolid pool’s longitudinal relaxation rate$R_{1}^{\text{s}}$.In this work, we quantify all parameters of the unconstrained 2-pool qMT model usinga tailored*hybrid-state*pulse sequence (Assländer, Mao, et al., 2024). Furthermore, due toprevious studies’ focus on clinically diagnosed MCI or AD (Duan et al., 2022;Giulietti et al., 2012;Makovac et al.,2018), it is unclear whether*preclinical*AD pathologycan be detected with qMT biomarkers. This distinction is important because Aβhas been shown to accumulate well before the clinical manifestations of dementia(Jack et al., 2010). Therefore, we focuson the preclinical population in this study. Our central hypothesis is that amyloidaccumulation in the preclinical population is detectable by qMTbiomarkers—including$R_{1}^{s}$—dueto the distinct biochemical properties of Aβ plaques.

## 2 Methods

### 2.1 Unconstrained magnetization transfer model

**Fig. 1.**
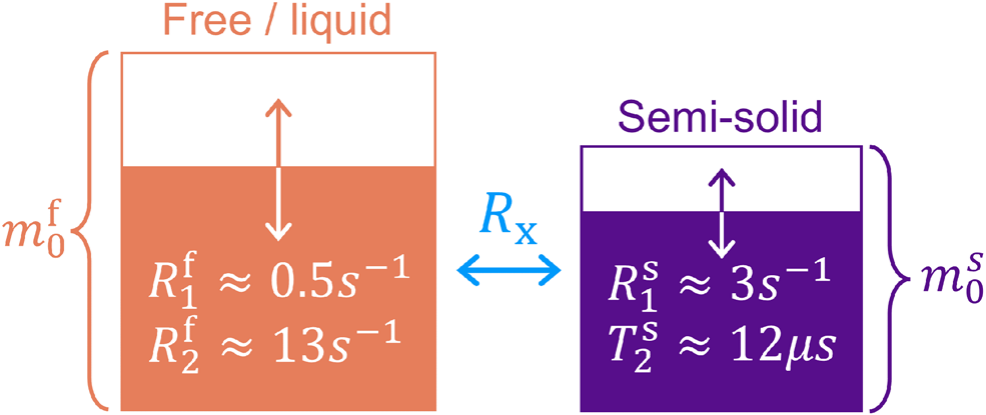
2-Pool quantitative magnetization transfer model (Assländer, Mao, et al., 2024;Henkelman et al., 1993). The redpool models “free water” protons with fraction$m_{0}^{f}$,while macromolecule-bound “semisolid” protons withfraction$m_{0}^{s}$are in purple($m_{0}^{s}+m_{0}^{f}=1$). Each pool’s longitudinal and transverserelaxation rates (reciprocal of the times)$R_{1}^{f,s}$and$R_{2}^{f,s}$, respectively, are modeled separately (most previousstudies constrain the value of$R_{1}^{s}$).After saturation of one pool’s longitudinal magnetization,relaxation and exchange with the other pool modulate each pool’smagnetization as visualized by the arrows and partially colored boxes.The exchange rate is denoted by$R_{x}$.

We use the unconstrained qMT model (Fig. 1)presented inAssländer, Mao, et al.(2024), which describes the Bloch–McConnell equations (McConnell, 1958) of the 2-pool spin system(Henkelman et al., 1993):

$$\partial_{t}\left[\begin{array}{c} x^{\text{f}}\\ y^{\text{f}}\\ \begin{array}{l} z^{\text{f}} \end{array}\\ x^{\text{s}}\\ z^{\text{s}}\\ 1 \end{array}\right]=\left[\begin{array}{cccccc} -R_{2}^{\text{f}} & -\omega_{z} & \omega_{y} & 0 & 0 & 0\\ \omega_{z} & -R_{2}^{\text{f}} & 0 & 0 & 0 & 0\\ -\omega_{y} & 0 & -R_{1}^{\text{f}}-R_{\text{x}}m_{0}^{\text{s}} & 0 & R_{\text{x}}m_{0}^{\text{f}} & m_{0}^{\text{f}}R_{1}^{\text{f}}\\ 0 & 0 & 0 & -R_{2}^{s,l}\left(T_{2}^{\text{s}},\alpha ,T_{RF}\right) & \omega_{y} & 0\\ 0 & 0 & R_{\text{x}}m_{0}^{\text{s}} & -\omega_{y} & -R_{1}^{\text{s}}-R_{\text{x}}m_{0}^{\text{f}} & m_{0}^{\text{s}}R_{1}^{\text{s}}\\ 0 & 0 & 0 & 0 & 0 & 0 \end{array}\right]\left[\begin{array}{c} x^{\text{f}}\\ y^{\text{f}}\\ \begin{array}{l} z^{\text{f}} \end{array}\\ x^{\text{s}}\\ z^{\text{s}}\\ 1 \end{array}\right],$$ (1)

where$\omega_{z}$is the off-resonance frequency and$\omega_{y}$is the Rabi frequency of the radiofrequency (RF) pulse which has a flip angleαand duration$T_{RF}$. The magnetization of protons bound in free water (superscript^f^) is described in Cartesian coordinates by$x^{\text{f}},y^{\text{f}},z^{\text{f}}$.The free pool’s magnetization exchanges at the rate$R_{\text{x}}$with the semisolid spin pool (superscript^s^), whose magnetization iscaptured in Cartesian coordinates by$x^{\text{s}},z^{\text{s}}$.Each pool has its own fractional size($m_{0}^{\text{f},\text{s}}$, where$m_{0}^{\text{f}}+m_{0}^{\text{s}}=1$), longitudinal($R_{1}^{\text{f},\text{s}}$) and transverse($R_{2}^{\text{f},\text{s}}$) relaxation rates (inverse of times$T_{1,2}$), amounting to six core parameters:$m_{0}^{\text{s}},\;\;R_{1}^{\text{f}},\;\;R_{2}^{\text{f}},\;\;R_{\text{x}},\;\;R_{1}^{\text{s}}$,and$T_{2}^{\text{s}}$.The nonexponential decay characteristics of the semisolid pool in thebrain’s parenchymal tissue are described by the super-Lorentzianlineshape (Morrison et al., 1995), wherewe use the time$T_{2}^{\text{s}}$(instead of the rate) for consistency with the qMT literature. These decaycharacteristics are incorporated in the Bloch–McConnell equation with thegeneralized Bloch model (Assländer et al.,2021). For computational efficiency, we approximate thenonexponential decay characteristics with an effective exponential decay usingthe*linearized*relaxation rate$R_{2}^{s,l}\left(R_{2}^{\text{s}},\alpha ,T_{RF}\right)$that results in the same magnetization at the end of an RF pulse with the flipangleαand pulse duration$T_{RF}$. Without loss of generality, we neglect$y^{\text{s}}$assuming$\omega_{x}\;=0$and$R_{2}^{s,l}\gg \omega_{z}$.More details regarding the generalized Bloch model are described inAssländer et al. (2021). Note theslight differences with respect to quantities often referenced in the MTliterature, including the pool-size ratio$\text{PSR}=\frac{m_{0}^{s}}{m_{0}^{f}}$(Henkelman et al., 1993), where$m_{0}^{s}=\frac{\text{PSR}}{1+\text{PSR}}$, and the “forward exchange rate,” which is theproduct$m_{0}^{s}\cdot R_{\text{x}}$.For the latter, many previous studies could not estimate the parameters$m_{0}^{\text{s}}$and$R_{\text{x}}$separately due to limitations of the employed encoding strategies and signalmodels (Ramani et al., 2002), which isovercome here by the use of a tailored hybrid-state pulse sequence.

The highly restricted motion of macromolecules leads to an ultrashort$T_{2}^{\text{s}}\approx 10\mu s$, which prevents direct detection of this pool with the typicalecho times achievable on clinical MRI scanners (i.e., without hardwaremodifications). Hence, the semisolid pool can be detected on clinical MRIscanners only indirectly via its exchange with the free pool, impeding theestimation of$R_{1}^{\text{s}}$.Consequently, authors have typically assumed$R_{1}^{\text{s}}\text{​}=1$s (Giulietti et al.,2012;Henkelman et al., 1993;Makovac et al., 2018). In this work,we overcome these limitations by utilizing a hybrid-state sequence (Assländer, Mao, et al., 2024;Assländer et al., 2019) (more detailsgiven inSection 2.4). We recentlydemonstrated*in vivo*the ability to voxel-wise quantify$R_{1}^{\text{s}}$(Assländer, Mao, et al., 2024),which takes on significantly smaller values than$R_{1}^{\text{f}}$(Helms & Hagberg, 2009;Manning et al., 2021;Samsonov & Field, 2021;van Gelderen et al., 2016). We hypothesized thateliminating this constraint on$R_{1}^{\text{s}}$increases qMT’s sensitivity to changes in the semisolid pool’sbiophysical properties arising from Aβ accumulation.

### 2.2 Comparison with constrained qMT

To test the above hypothesis, we compare the unconstrained qMT parameters withtheir equivalents in the constrained qMT model. Specifically, we compute theapparent macromolecular pool size$m_{0}^{\text{s},\text{a}}$as

$$m_{0}^{\text{s},\text{a}}\;=m_{0}^{\text{s}}\left(1-\frac{2m_{0}^{\text{f}}\left(R_{1}^{\text{s}}-R_{1}^{\text{f}}\right)}{R_{\text{x}}}\right),$$ (2)

the apparent magnetization exchange rate$R_{\text{x}}^{\text{a}}$as

$$R_{\text{x}}^{\text{a}}=\left(R_{\text{x}}\text{​}+R_{1}^{\text{f}}\right)+m_{0}^{\text{f}}\left(R_{1}^{\text{s}}-R_{1}^{\text{f}}\right)+\frac{m_{0}^{\text{f}}m_{0}^{\text{s}}{\left(R_{1}^{\text{s}}-R_{1}^{\text{f}}\right)}^{2}}{R_{\text{x}}},$$ (3)

and the apparent longitudinal relaxation rate$R_{1}^{\text{f},\text{a}}$as

$$R_{1}^{\text{f},\text{a}}=R_{1}^{\text{f}}+m_{0}^{\text{s}}\left(R_{1}^{\text{s}}-R_{1}^{\text{f}}\right)-\frac{m_{0}^{\text{f}}m_{0}^{\text{s}}{\left(R_{1}^{\text{s}}-R_{1}^{\text{f}}\right)}^{2}}{R_{\text{x}}}.$$ (4)

Full details regarding these expressions for the constrained qMT model can befound inSection 2.2ofAssländer, Mao, et al. (2024).

### 2.3 Study participants

We recruited 15 cognitively normal participants (with a Clinical DementiaRating^®^of 0) from New York University’s ADResearch Center (ADRC) cohort of community-dwelling elderly adults. Theassessment of being cognitively normal was based on a comprehensive set ofpsychometric tests across multiple cognitive domains that informed a consensusdiagnosis. Hence, the yearly conversion rate to MCI is extremely low in thiscohort within the NYU ADRC. All 15 participants had received amyloid PET scanswithin the preceding 34 months (mean±standard deviation 17±9 months). Six individuals were consideredAβ−by our ADRC’s standardized uptake value ratio (SUVR) threshold (seeSection 2.8), with the followingdemographics: three female, three white, age 72.6±4.5 years (mean±standard deviation), Montreal Cognitive Assessment (MoCA) scores 27.3±1.2, one*APOE*-ε4 and two*APOE*-ε2carriers. The remaining nine participants wereAβ+,with the following demographics: five female, seven white, age 75.6±6.5 years, MoCA 27±2.2, and six*APOE*-ε4 carriers. All MoCA scores weretaken from each participant’s annual ADRC visit that was closest in timeto the date of the qMT scan. With the exception of oneAβ+participant, all MoCA scores were obtained within a 10-month period eitherpreceding or following the respective qMT scan. All participants providedwritten informed consent for the studies described below, in agreement with therequirements of the New York University School of Medicine Institutional ReviewBoard.

### 2.4 Imaging protocol

All participants received 300 MBq (8.1 mCi) of [^18^F]florbetaben (FBB)tracer (Life Molecular Imaging, Totowa, NJ) intravenously over 15 s, followed bya 12-cc saline flush. Syringes were assayed pre- and postinjection. Participantsrested in the injection room to achieve brain equilibration of the tracer beforebeing positioned in the scanner. Amyloid PET scans were acquired on a 3 TeslaBiograph mMR PET/MRI system (Siemens Healthineers, Germany) 90–120 minpostinjection. Structural MRI was also obtained for registration to images fromthe next session.

In a second session 17±9 months (no more than 34 months) later, each participant underwent a 24-min MRIexamination on a 3 Tesla Prisma MRI scanner (Siemens Healthineers, Germany)using a 32-channel head coil. Our experimental whole-brain qMT technique used ahybrid-state sequence (Assländer et al.,2019) optimized for quantifying MT parameters with a nominal 1 mmisotropic resolution (effectively 1.24 mm isotropic accounting for 3D radialkoosh-ball sampling of only the in-sphere of a 1 mm k-space cube) across a 256×256×256 mm FOV in 12 min (Assländer, Mao, etal., 2024). The hybrid-state sequence is similar to aninversion-recovery balanced steady-state free precession (bSSFP) sequence (Bieri & Scheffler, 2013;Carr, 1958) in that it uses fully balancedgradient moments per repetition time, but it also incorporates a smoothlyvarying flip angle and RF pulse duration between repetition times (Assländer et al., 2019) that wasoptimized for the encoding of the qMT parameters (Assländer, Mao, et al., 2024). However, we note that the RFpattern was optimized for measuring demyelination in white matter rather thantailored specifically for the detection of Aβ in the cortex. k-Spacereadout was performed with a radial koosh ball trajectory, where the directionof the 3D radial spokes was distributed across the unit sphere using a 2D goldenmeans pattern (Chan et al., 2009),reshuffled to minimize eddy current artifacts (Flassbeck et al., 2024). More details about the hybrid-statesequence—including the encoding mechanisms and its numerical optimizationfor quantifying the unconstrained qMT parameters—can be found inAssländer, Mao, et al. (2024).

3D Magnetization-Prepared Rapid Acquisition Gradient-Recalled Echo (MPRAGE)(Brant-Zawadzki et al., 1992;Mugler & Brookeman, 1990) andT2-weighted Fluid-Attenuated Inversion-Recovery (FLAIR) (Hajnal et al., 1992) images were also acquired with 1mm isotropic resolution. Both sequences were GRAPPA 2x accelerated, where theMPRAGE used a TE/TR/TI (echo time, repetition time, and inversion time) of 2.98ms/2.3 s/900 ms for 5 min 30 s of scan time and the FLAIR used a TE/TR/TI of 392ms/5 s/1.8 s for 5 min 57 s of scan time.

### 2.5 Image reconstruction

PET reconstruction used the Standardized Centralized Alzheimer’s &Related Dementias Neuroimaging (SCAN) parameters (https://scan.naccdata.org),except modified to use only a single frame of the first 10 min of data to reducemotion-related artifacts (Koesters et al.,2016): OSEM-3D (Erdogan &Fessler, 1999) with 4 iterations and 21 subsets; 344×344×127 grid; 2.0 zoom (1.04313×1.04313×2.03125 mm voxels); all corrections on; postreconstruction smoothing with a 2 mmfull width at half maximum Gaussian kernel. Attenuation correction was performedusing MR-based hybrid segmentation and atlas-based algorithm that combinestissue segmentation from a Dixon µ-map with a superimposed, coregistered,skull atlas-derived bone compartment (Koesterset al., 2016).

For the qMT sequence, we performed self-navigated motion correctionretrospectively with a temporal resolution of 4 s based on low-resolutionreconstructions (4 mm isotropic) in a subspace optimized for contrast betweenthe parenchyma and CSF (Flassbeck et al.,2024;Kurzawski et al., 2020).From the corrected k-space data, we reconstructed 15 coefficient imagescorresponding to a low-rank representation of the MRI signal’s temporaldynamics using a subspace modeling approach (Assländer et al., 2018;Liang, 2007;McGivney et al.,2014;Tamir et al., 2017). Thesubspace was spanned by singular vectors computed from a dictionary of signals(or fingerprints) and, additionally, their orthogonalized gradients, whichmaximizes the information needed to stably perform parameter estimation forcomplex pulse sequences (Mao, Flassbeck,Gultekin, & Assländer, 2024). The reconstruction alsoutilized sensitivity encoding (Pruessmann etal., 2001;Sodickson &Manning, 1997), with coil sensitivities estimated using ESPIRiT(Uecker et al., 2014), and locallylow-rank regularization to minimize undersampling artifacts and noise (Lustig et al., 2007;Trzasko & Manduca, 2011;T. Zhang et al., 2015). The reconstruction wasimplemented using the optimal iterative soft thresholding algorithm (Jang et al., 2023) in*Julia*based on publicly available source code (seeSection 5). More details about thereconstruction can be found inTamir et al.(2017)andAssländer et al.(2018).

### 2.6 qMT model fitting

**Fig. 2.**
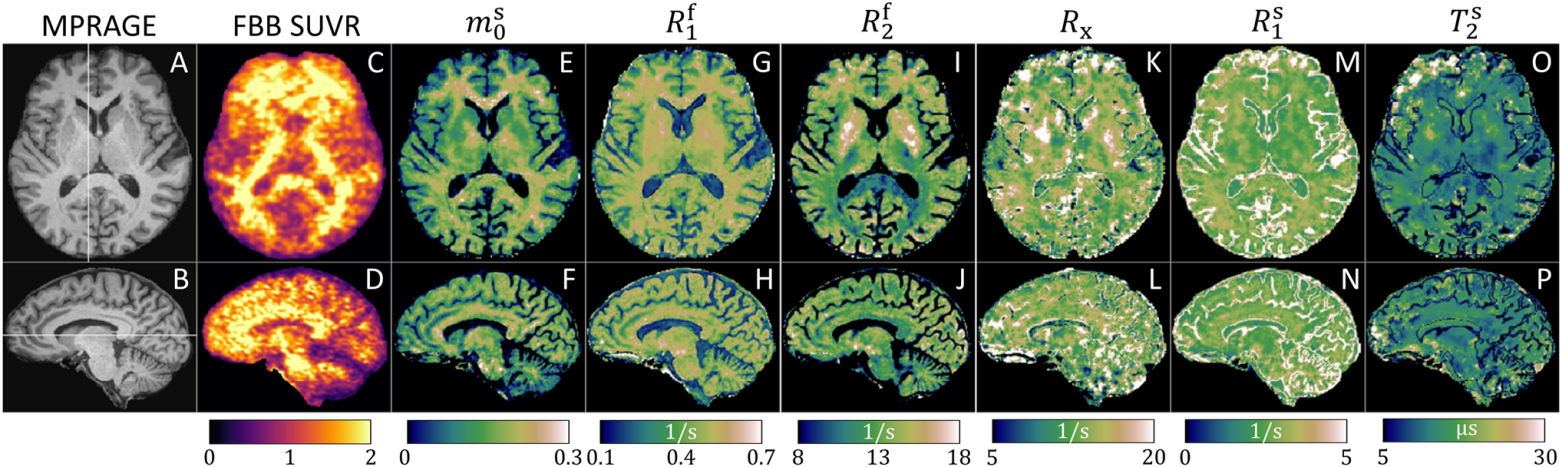
Exemplary MPRAGE (A–B), [^18^F]florbetaben (FBB) PET SUVR(C–D), and quantitative magnetization transfer maps (E–P)in a cognitively normalAβ+participant. The six qMT parameters are the semisolid pool size$m_{0}^{s}$,the relaxation rates$R_{1,2}^{\text{f},\text{s}}=1\text{​}/T_{1,2}^{\text{f},\text{s}}$(where the superscripts^f^and^s^denote the free and semisolid spin pools, respectively), and theexchange rate$R_{\text{x}}$. The MPRAGE is used for FreeSurfer-based corticalparcellation and calculation of cortical thicknesses. The FBB SUVRimages are used to compare the qMT parameters with a relativeconcentration of Aβ.

Following image reconstruction, we estimated maps of the six qMT parameters($m_{0}^{\text{s}},\;\;R_{1}^{\text{f}},\;\;R_{2}^{\text{f}},\;\;R_{\text{x}},\;\;R_{1}^{\text{s}}$,and$T_{2}^{\text{s}}$)by voxel-wise fitting the unconstrained qMT model to the reconstructedcoefficient images. For computational efficiency, we used a neural network-basedestimator. This network, tailored for our sequence, takes the 15 complex-valuedcoefficients, split into real and imaginary parts, as inputs. The architecturewas similar to the design described in Figure 2 ofX. Zhang et al. (2022): 11 fully connected layers(with layer widths30→256→512→1024→768→512→384→256→128→30→32→6) with batch normalization, skip connections, and RectifiedLinear Unit activation functions (seeSection5for code; however, we note that the specific architecture employedis not essential in determining the neural network’s performance). Thenetwork incorporated a data-driven correction for$B_{0}$and$B_{1}^{+}$inhomogeneities as described inAssländer,Gultekin, et al. (2024), and was trained explicitly to minimize thebias that is typically introduced when assuming a distribution for the simulatedtraining data (Mao, Flassbeck, &Assländer, 2024). Example parameter maps for anAβ+participant are shown inFigure 2.

### 2.7 Image processing

The MPRAGE, FLAIR, and FBB images were skull stripped (Hoopes et al., 2022) and rigid-body registered (Reuter et al., 2010) to the estimated$m_{0}^{\text{s}}$maps (which has the most similar contrast to the MPRAGE among the qMTparameters) using the “mri_synthstrip” and“robust_register” commands, respectively, in FreeSurfer v7.4.1(Fischl, 2012). The qMT parameterswere chosen as the common reference frame to avoid interpolating the nonlinearlyprocessed qMT maps. Cortical and subcortical segmentation, corticalparcellation, volume, and thickness values were then computed usingFreeSurfer’s “recon-all” command (Fischl, 2004;Fischlet al., 2002). Volumes were normalized by the estimated totalintracranial volume, and the average global cerebellar FBB value was used fornormalization to compute SUVRs.

Because the cortices are only 2–3 mm thick compared with the 1.24 mmeffective isotropic resolution, and since the quantification of the several qMTparameters is unstable in the CSF (where the macromolecular pool size$m_{0}^{\text{s}}$approaches zero), cortical analyses of our qMT parameter maps are highlysusceptible to partial volume effects. To mitigate this issue, we adopt aconservative approach of sampling the qMT and FBB SUVR values at the surfacecorresponding to 50% of the cortical depth using FreeSurfer’s“mri_vol2surf” function. To avoid resampling the nonlinearlyprocessed qMT data and minimize linear interpolation error when using theFreeSurfer tools, we applied the following procedure. First, we sincinterpolated (Oppenheim & Willsky,1996) the reconstructed coefficient images onto a 5×finer grid (i.e., 0.2×0.2×0.2mm voxels). Then, we used “mri_vol2surf” with trilinearinterpolation to sample the coefficient images at 50% of the cortical depthbefore applying the neural network to estimate the qMT parameter maps on theinterpolated surface. We note that this approach still depends on the accuracyof FreeSurfer’s estimated gray and white matter surfaces.

### 2.8 Standardized uptake value ratio calculation

The amyloid SUVR positivity threshold of 1.08 was determined by the NYU ADResearch Center through an image processing procedure on their entire cohort ofparticipants who have received florbetaben PET scans since 2021, independent ofthe analyses performed in this manuscript. According to the recommended ADNImethods (Landau et al., 2021),FreeSurfer-derived Desikan–Killiany atlas regions were used to constructa composite neocortical ROI comprising the frontal, lateral parietal, andlateral temporal cortex as the “target” region, where the wholecerebellum served as the “reference” region. The neocortical SUVRwas computed based on the mean activity within.

### 2.9 Lobar analysis

Due to small sample sizes, we perform a lobar-level analysis by grouping theDesikan–Killiany cortical ROIs (Desikanet al., 2006) into the four primary cortical lobes (frontal,parietal, temporal, and occipital) using the schema suggested in the Appendix ofKlein and Tourville (2012). Eachparticipant’s average measurement (qMT parameter, FBB SUVR, or thickness)per cortical lobe and hemisphere is estimated from the constituent ROIs using aninverse-variance weighting (Kay, 1993)based on each ROI’s sample variance as computed by FreeSurfer’s“mri_segstats” function (i.e., the maximum likelihood estimateassuming the “mri_vol2surf” samples from each cortical ROI arenormally distributed). Prior to all statistical analyses, we manually excludedfour ROIs (for all measurements) where we identified substantial artifacts in$R_{\text{x}}$across multiple participants, likely caused by subcutaneous fat (seeSection 4.2): the rostral anteriorcingulate, precentral, postcentral, and superior parietal gyri. We also excludedthree ROIs (for all measurements) superior to the frontal sinus that exhibitedbSSFP-like banding artifacts (Bieri &Scheffler, 2013) in the qMT parameters (Fig. 2L): the medial and lateral orbitofrontal cortices and temporalpole.

We also consider a synthetic lobe based on the “signature of AD-relatedcortical thinning” described inDickersonet al. (2011), which defines a group of cortical regions identifiedas being the most vulnerable to thinning in a large cohort of cognitively normalindividuals who later developed AD dementia. From the Desikan–Killianyatlas, we chose the following ROIs that most closely matched those described inDickerson et al. (2011)to computethe AD-signature summary measure: the entorhinal cortex, temporal pole, inferiortemporal gyrus, angular gyrus, supramarginal gyrus, superior parietal cortex,precuneus, middle frontal gyrus, and superior frontal gyrus.

### 2.10 Subcortical analysis

As a secondary outcome of our study, we also computed average measurements insubcortical structures based on the FreeSurfer segmentation: the hippocampus,amygdala, thalamus, caudate, putamen, and pallidum.

### 2.11 Statistical analysis

We used the nonparametric Mann–WhitneyUtest (Fay & Proschan, 2010) tocompare measurements between theAβ−andAβ+groups. We considered thep<0.05, Bonferroni-correctedp<0.01(accounting for the five cortical “lobes”considered), andp<0.007(accounting for six subcortical and global WM ROIs)significance levels, where the latter two were used to account for thefamily-wise error rate (Dunn, 1961) inthe group comparisons shown inFigures3–5. Effect sizes were quantified using Hedge’sg(Hedges, 1981), where we considered0≤|g|<0.5“small,”0.5≤|g|<0.8“medium,”0.8≤|g|<1.2“large,” and|g|≥1.2“very large” (Sawilowsky,2009). A positive effect size (g>0) wasdefined as measurements that were larger in theAβ+group. We used Pearson’s correlation coefficient to evaluate theassociation of our measurements with amyloid burden, again using thep<0.05significance level. All statistical analyses were performedusing the*HypothesisTests.jl Julia*package.

**Fig. 3.**
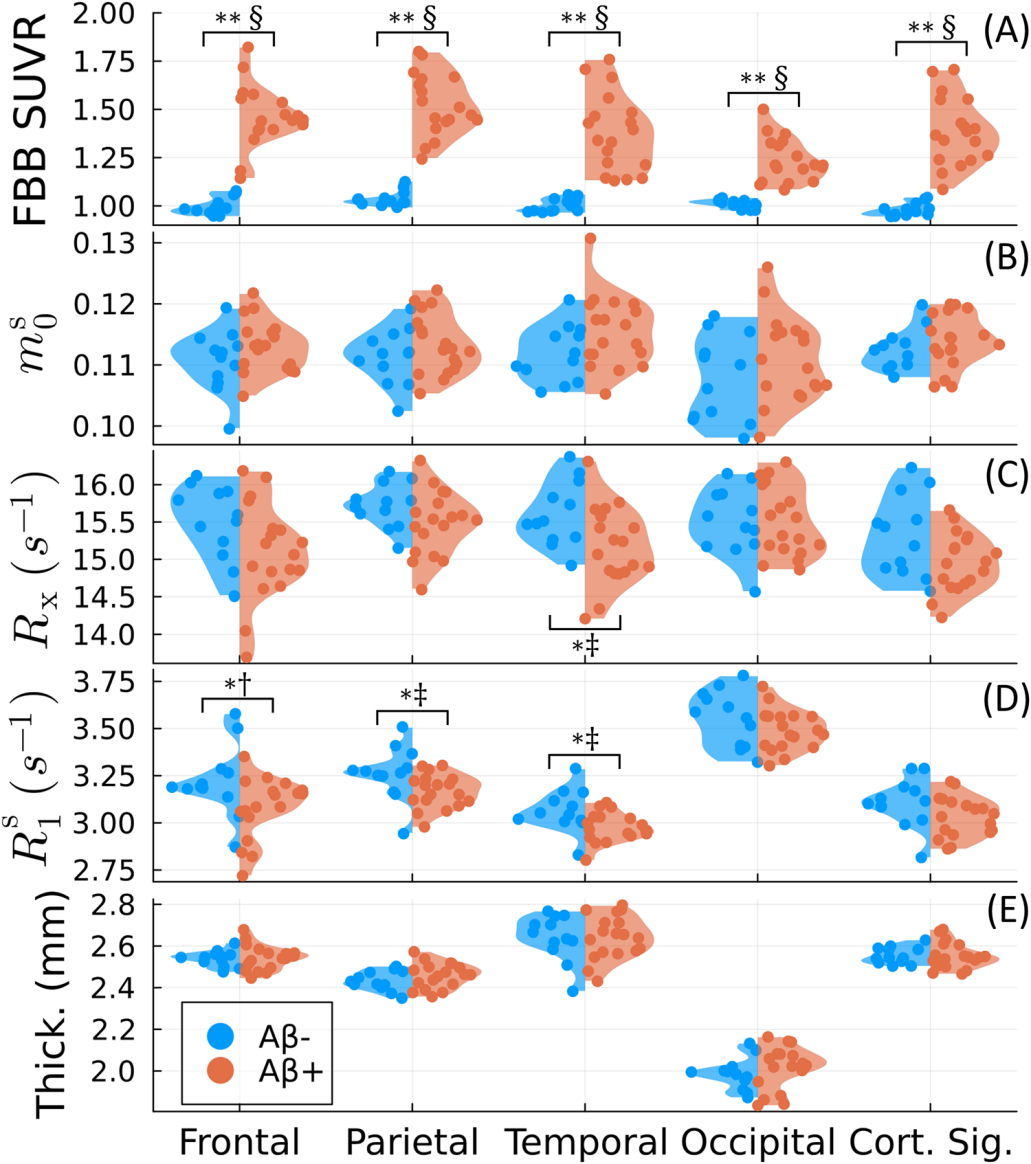
Group comparison for the four cortical lobes and the “corticalsignature of thinning” (Dickersonet al., 2011). [^18^F]florbetaben (FBB) SUVR (A),three qMT parameters (the macromolecular pool size$m_{0}^{\text{s}}$(B), the magnetization exchange rate$R_{\text{x}}$(C), and the macromolecular pool’s longitudinal relaxation rate$R_{1}^{\text{s}}$(D)), and cortical thickness (E) are compared between amyloid negative(Aβ−)and positive(Aβ+)groups. Each dot represents the average lobar value per cerebralhemisphere for each of the 15 participants.*and**denote statistical significance forp<0.05and the Bonferroni-correctedp<0.01, respectively, using the Mann–WhitneyUtest (Fay & Proschan,2010). †, ‡, and § indicate medium, large,and very large effect sizes using Hedge’sg(Hedges, 1981;Sawilowsky, 2009), respectively.Thepandgvalues are summarized in SupportingTable S1andgiven for the remaining qMT parameters in SupportingTable S2.

## 3 Results

Figure 3compares the qMT parameters, FBB SUVR,and cortical thickness values between theAβ−andAβ+groups. Uniformly across the neocortex, SUVR values were significantly increased inAβ+participants with very large effect sizes, which was expected from our definition ofamyloid positivity. Consistently with previous literature reports, the magnetizationexchange rate$R_{\text{x}}$was significantly decreased in the temporal lobe with a large effect size (g=−0.86). The macromolecular pool’s longitudinal relaxation rate$R_{1}^{\text{s}}$was also significantly decreased in the frontal, parietal, and temporal lobes withmedium (g=−0.78), large (g=−0.81), and large (g=−0.94) effect sizes, respectively. By contrast, no significant groupdifferences were observed in any lobe for the macromolecular pool size$m_{0}^{\text{s}}$,cortical thickness (including for the “AD cortical signaturemeasure”), or the remaining qMT parameters (shown in SupportingTable S2).

**Fig. 4.**
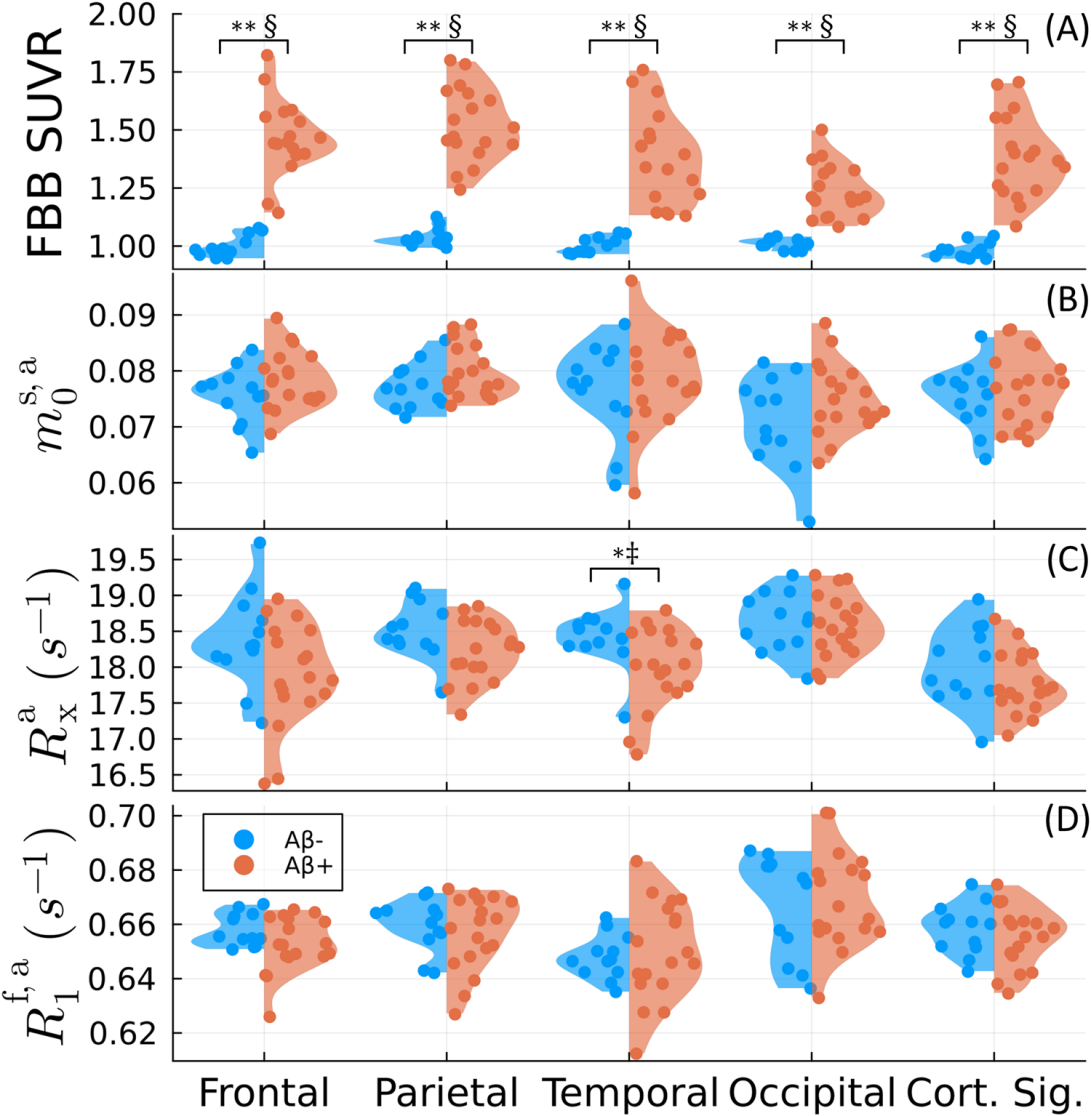
Repetition of the group comparison inFigure3with the established qMT model that is constrained by$R_{1}^{\text{s}}=R_{1}^{\text{f}}$.The constrained model parameters are approximated from their unconstrainedcounterparts as described inAssländer, Mao, et al. (2024)on a voxel basis. (A)[^18^F]florbetaben (FBB) SUVR, (B) the apparent macromolecularpool size$m_{0}^{\text{s},\text{a}}$, (C) the apparent magnetization exchange rate$R_{\text{x}}^{\text{a}}$,and (D) the apparent longitudinal relaxation rate of the free pool$R_{1}^{\text{f},\text{a}}$.*and**denote statistical significance forp<0.05and the Bonferroni-correctedp<0.01, respectively, using the Mann–WhitneyUtest (Fay & Proschan, 2010).‡ and § indicate large and very large effect sizes usingHedge’sg(Hedges, 1981;Sawilowsky, 2009), respectively.

Figure 4shows the group comparison using the*constrained*qMT model described inSection 2.2. Here, the apparent magnetization exchange rate$R_{\text{x}}^{\text{a}}$is still significantly decreased in the temporal lobe, which isconsistent with previous literature reports that also assume constraints on thevalue of$R_{1}^{\text{s}}$(Giulietti et al., 2012;Makovac et al., 2018). However,$R_{1}^{\text{f},\text{a}}$does not exhibit significant group differences in any corticallobe, unlike the unconstrained$R_{1}^{\text{s}}$.This analysis suggests increased information coupling between the qMT parameters inthe constrained model that reduces the overall sensitivity to amyloid burden.

We repeated the analysis using the unconstrained model for the FreeSurfer-definedglobal white matter and subcortical structures, shown inFigure 5and SupportingTables S3–S4. There were no significant differences inthe volumes of any subcortical structures (including the hippocampus (Sabuncu et al., 2011)) or total CSF (notshown). However, we observed significant decreases in both free pool relaxationtimes ($R_{1}^{\text{f}}$and$R_{2}^{\text{f}}$)in the global white matter, hippocampus, and thalamus.$m_{0}^{\text{s}}$and$R_{1}^{\text{f}}$were also significantly decreased in the pallidum.

**Fig. 5.**
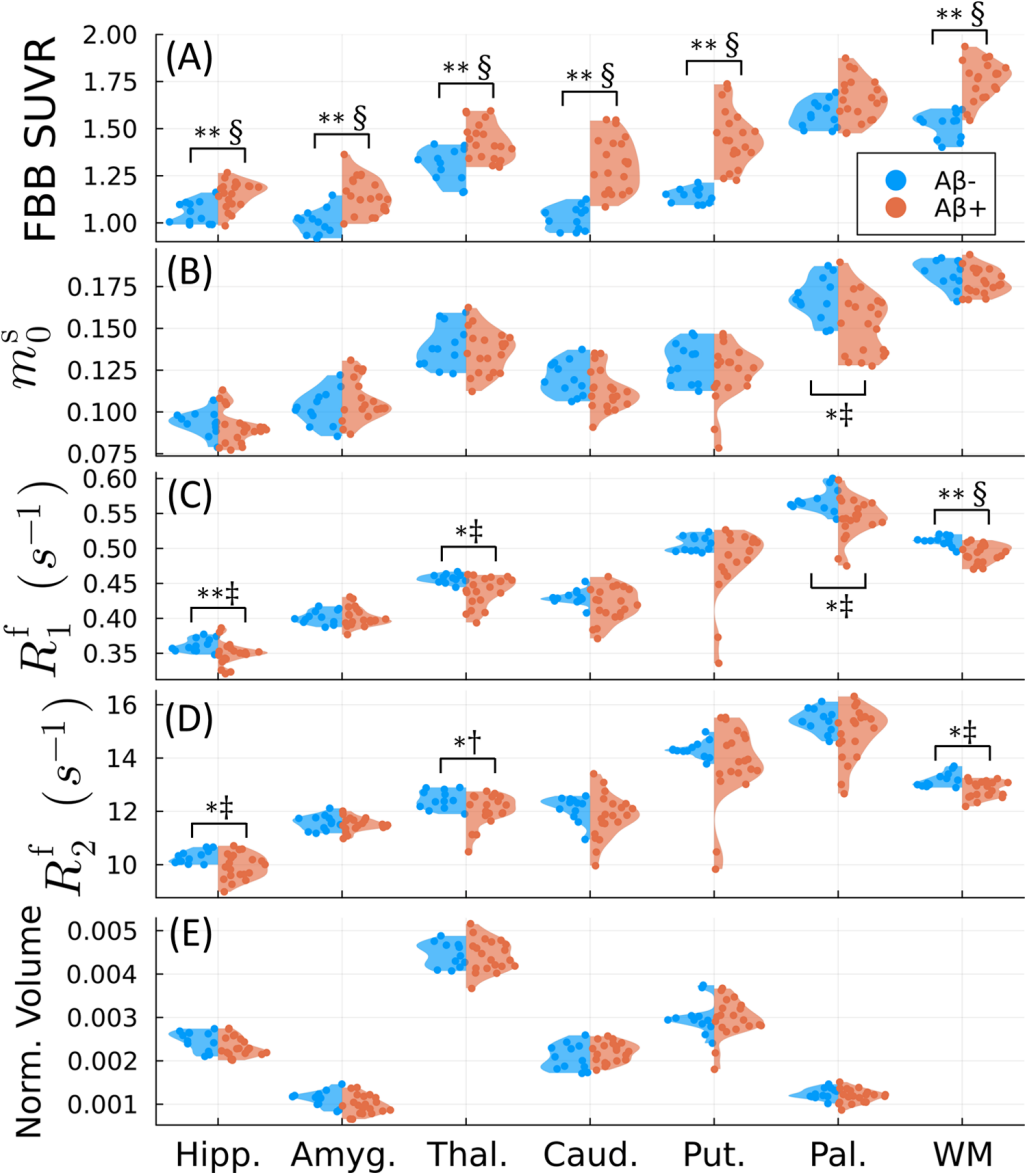
Group comparison for subcortical (hippocampus, amygdala, thalamus, caudate,putamen, pallidum) and global white matter (WM) ROIs.[^18^F]florbetaben (FBB) SUVR (A), three qMT parameters (themacromolecular pool size$m_{0}^{\text{s}}$(B), the free pool’s longitudinal relaxation rate$R_{1}^{\text{f}}$(C), and the free pool’s transverse relaxation rate$R_{2}^{\text{f}}$(D)), and volumes (E, normalized by the estimated total intracranial volume)are compared between amyloid negative(Aβ−)and positive(Aβ+)groups. Each dot represents the average lobar value per cerebral hemispherefor each of the 15 participants. Note that the global white matter volumescould not be visualized within the plotted axes, but there were nosignificant group differences.*and**denote statistical significance forp<0.05and the Bonferroni-correctedp<0.007, respectively, using the Mann–WhitneyUtest (Fay & Proschan, 2010).†, ‡, and § indicate medium, large, and very largeeffect sizes using Hedge’sg(Hedges, 1981;Sawilowsky, 2009), respectively. Thepandgvalues are summarized in SupportingTable S3and given for the remaining qMT parametersin SupportingTableS4.

**Fig. 6.**
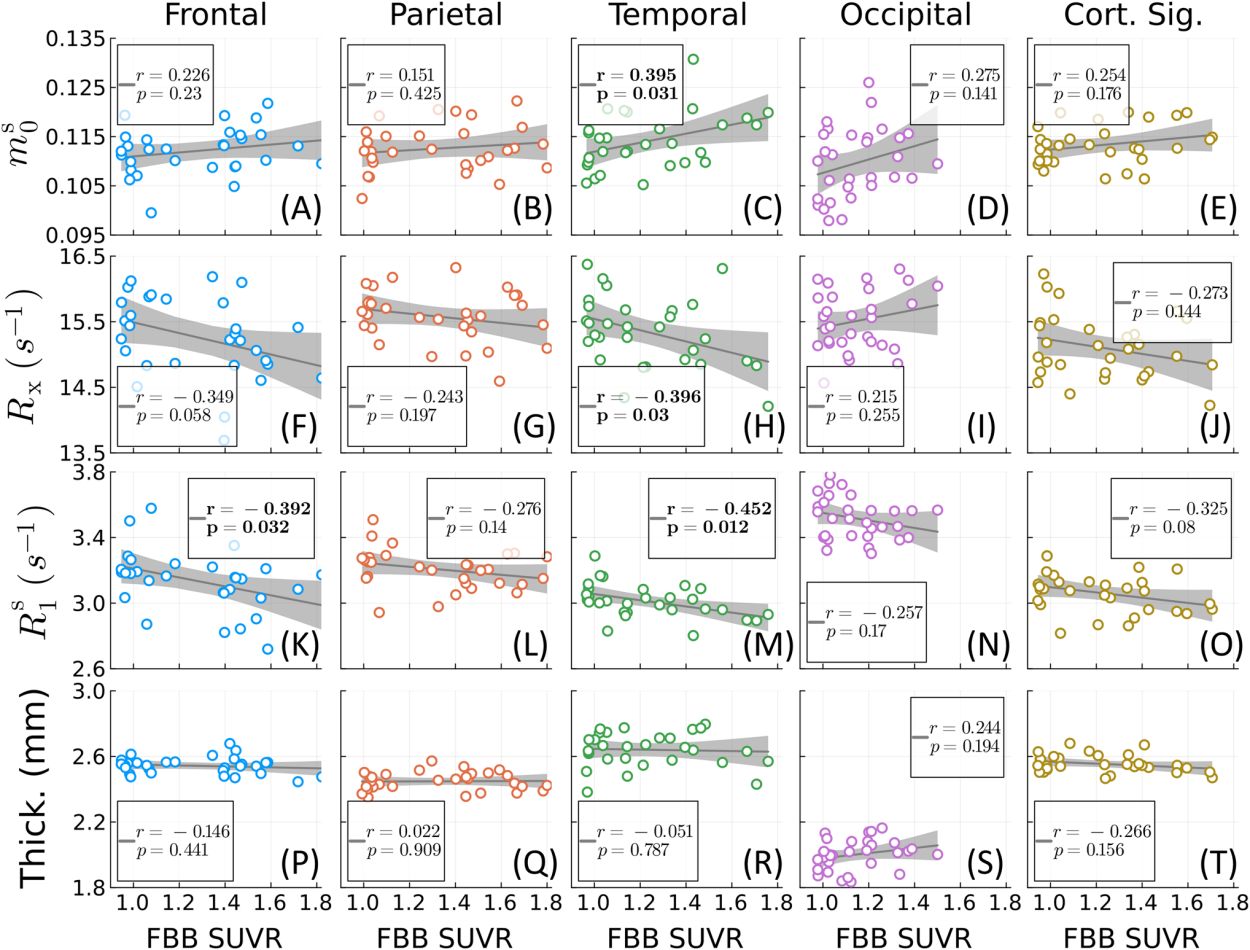
Correlation analysis for the four cortical lobes and the “corticalsignature of thinning” (Dickerson etal., 2011). Three qMT parameters—the macromolecular poolsize$m_{0}^{\text{s}}$(A–E), the magnetization exchange rate$R_{\text{x}}$(F–J), and the macromolecular pool’s longitudinal relaxationrate$R_{1}^{\text{s}}$(K–O)—and cortical thickness (P–T) are plotted againstamyloid burden as measured by [^18^F]florbetaben (FBB) PET SUVR.Each dot represents the average lobar value per cerebral hemisphere for eachof the 15 participants. Significant Pearson’s correlations (p<0.05)are bolded.

Figure 6analyzes the correlation between theunconstrained qMT parameters or cortical thickness and amyloid concentration (asmeasured by FBB PET SUVR). Significant Pearson’s correlations occur inlocations similar toFigure 3, though with afew differences: there was no significant negative correlation with$R_{1}^{\text{s}}$in the parietal lobe, but a significant positive correlation with$m_{0}^{\text{s}}$in the temporal lobe. The joint consistency betweenFigures 3and6improves theoverall confidence in the results. For example, while the significant decrease in$R_{\text{x}}$within the temporal lobe inFigure 3appears tobe driven primarily by a couple of participants,Figure 6demonstrates that there is also a significant trend towarddecreased values as a function of amyloid concentration. Similarly toFigure 3, no significant correlations wereobserved between cortical thickness and amyloid concentration in any lobe (neitherfor the “AD cortical signature measure”). Overall, the direction ofcorrelation (positive or negative) for each measure is the same across all lobesexcept in the occipital lobe for$R_{\text{x}}$.We note, however, that this outlier is not statistically significant.

To understand the spatial correspondence between the unconstrained qMT measurementsand FBB SUVR, we visualize the corresponding effect sizes for each cortical ROIoverlaid on the Desikan–Killiany atlas inFigure 7. Note that because increases in FBB SUVR as opposed todecreases in$R_{\text{x}}$/$R_{1}^{\text{s}}$were observed in theAβ+group inFigures 3and6, a reversed color bar is used for FBB SUVR for ease ofcomparison across the different measurements. As expected, very large positiveeffect sizes are uniformly observed across the cortex for FBB SUVR. While we do notobserve uniformly large*negative*effect sizes for$R_{\text{x}}$and$R_{1}^{\text{s}}$,the ROIs exhibiting (likely spurious)*positive*effects somewhatoverlap with the ROIs excluded from the lobar analyses for exhibiting imagingartifacts (e.g., the postcentral and superior parietal gyri in$R_{\text{x}}$).Importantly, though we do observe small (g=−0.42) to medium (g=−0.69) effects suggesting subtle thinning of the right/left entorhinalcortices (Dickerson et al., 2011;Sabuncu et al., 2011), the qMT parametersexhibit higher overall sensitivity to amyloid burden across the entireneocortex.

**Fig. 7.**
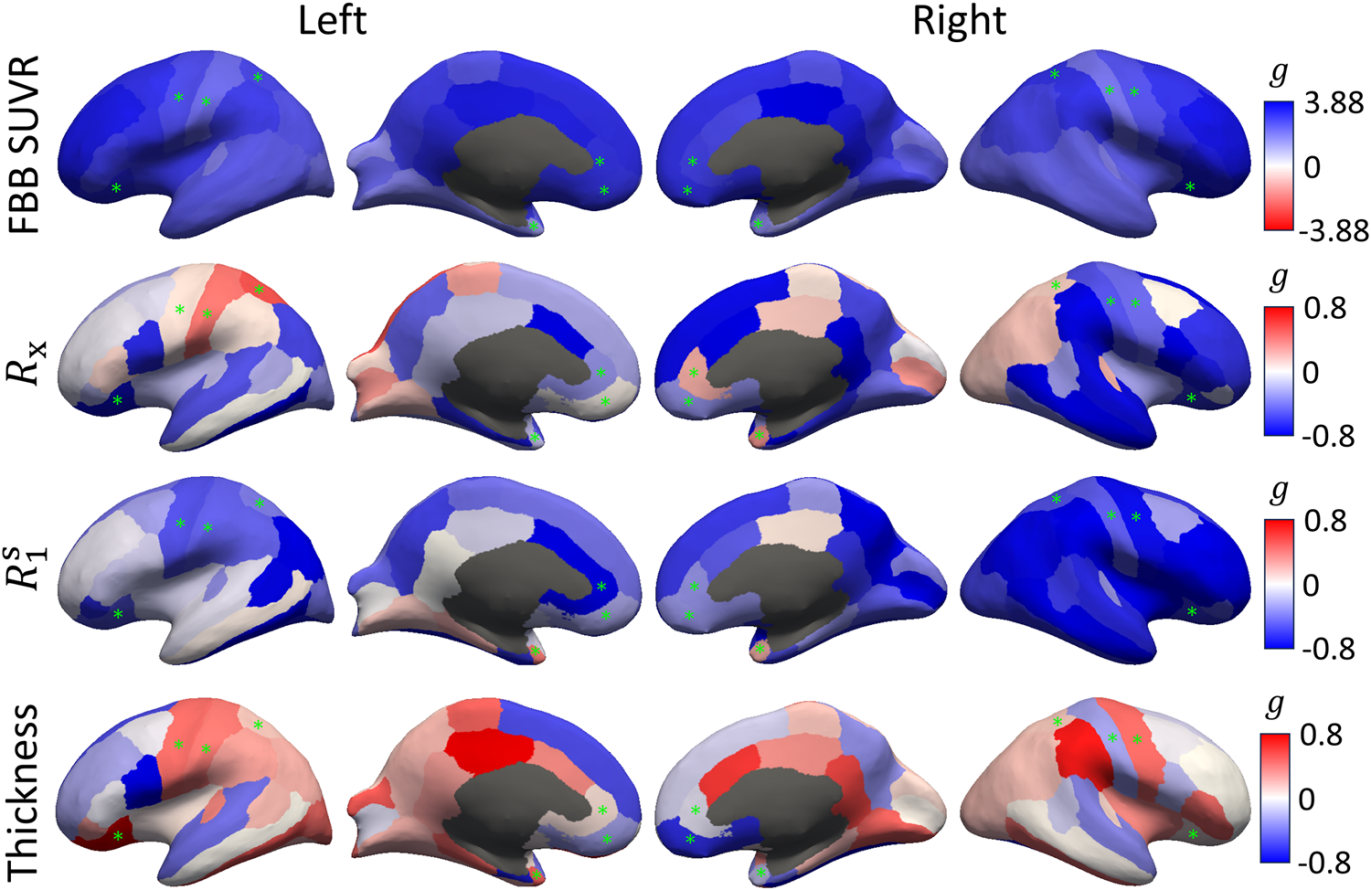
Effect sizes across the neocortex. Hedge’sg(Hedges, 1981) comparingAβ−andAβ+groups is overlaid on the Desikan–Killiany surface atlas (Desikan et al., 2006) for themagnetization exchange rate$R_{\text{x}}$,the macromolecular pool’s longitudinal relaxation rate$R_{1}^{\text{s}}$,and cortical thickness in comparison with [^18^F]florbetaben (FBB)PET SUVR. A positive effect size (g>0) means measurements are larger in theAβ+group, and|g|=0.8(a “large” effect (Sawilowsky, 2009)) is used as an illustrativecutoff. For FBB SUVR, note the modified cutoffs using the maximal|g|value (due to very large positive effect sizes) and the reversed color bar,used to color the expected direction of effects as blue for all measures(positive for FBB SUVR, negative for$R_{\text{x}}$,$R_{1}^{\text{s}}$,and thickness). The green asterisks denote ROIs excluded from thelobar-level analyses for all measures inFigures 3–6.

## 4 Discussion

Our study revealed widespread group differences betweenAβ+andAβ−individuals across the various unconstrained qMT parameters. The largest effect wasobserved in the semisolid pool’s longitudinal relaxation rate$R_{1}^{\text{s}}$which, notably, is not detectable in any of the constrained qMT parameters (cf.Fig. 4).$R_{1}^{\text{s}}$was previously considered inaccessible and typically constrained. However, using thehybrid-state’s enhanced signal encoding capabilities (Assländer, Gultekin, et al., 2024;Assländer, Mao, et al., 2024;Assländer et al., 2019), we are able to quantify$R_{1}^{\text{s}}$on a voxel-by-voxel basis. Our data suggest that$R_{1}^{\text{s}}$may be a potential biomarker for amyloid pathology preceding morphometric measuresof atrophy (Dickerson et al., 2011;Sabuncu et al., 2011). A potential biophysicalexplanation for this finding could be the slower longitudinal relaxation of spinsbound specifically in Aβ plaques relative to other constituents of themacromolecular pool, such as myelin.

We also observed a reduction in$R_{\text{x}}$inAβ+participants, which has previously been attributed to the hydrophobicity ofAβ plaques (Chen et al., 2017;Giulietti et al., 2012). This finding alignswith previous reports that the forward exchange rate (i.e.,$m_{0}^{\text{s}}\cdot R_{\text{x}}$)is reduced in*clinical*AD and is predictive of amnestic MCI to ADconversion (Duan et al., 2022;Giulietti et al., 2012;Makovac et al., 2018). Our results using an*unconstrained*qMT model, however, suggest that$R_{\text{x}}$is more sensitive to amyloid pathology than$m_{0}^{\text{s}}$in*preclinical*AD. This finding raises an interesting possibility:if neurodegeneration causes reduced$m_{0}^{\text{s}}$in advanced disease (discussed further inSection4.2),$R_{\text{x}}$and$m_{0}^{\text{s}}$may each potentially be associated with the “A” and “N”axes of the A/T/N framework (Jack et al.,2018), respectively. This emphasizes the importance of separating theseparameters in the unconstrained qMT model.

Contrary to our expectations of an increase in the macromolecular pool size$m_{0}^{\text{s}}$corresponding with greater amyloid burden, we found no significant group differencesin$m_{0}^{\text{s}}$,although a positive correlation was observed in the temporal lobe. One explanationcould be a competing effect causing a simultaneous decrease in$m_{0}^{\text{s}}$,possibly due to concomitant neurodegeneration (preceding a macroscopic change incortical thickness) or a change in the interstitial load of water (for example, dueto reduced fluid clearance (Tarasoff-Conway et al.,2015) or vascular leakage from reactive astrogliosis (Nakahara et al., 1999)). However, the latter hypothesisappears less likely given the lack of significant changes we would expect in thefree water pool’s relaxation rates$R_{1}^{\text{f}}$and$R_{2}^{\text{f}}$(Assländer, Mao, et al., 2024;Stanisz et al., 2004).

### 4.1 Limitations

Our preliminary study was designed to assess the utility of unconstrained qMTbiomarkers for detecting Aβ accumulation and has several limitations.Firstly, a larger cohort is needed to verify our findings, control for theeffect of nuisance variables (e.g., age, sex, race), and increase thestatistical power to perform ROI or voxel-level analyses. Secondly, the temporaldelay between the acquisition of the amyloid PET and qMT scans introduces somebias which limits the power of our study to detect group differences. WhileSupportingFigure S1shows that there is no significant correlation between the interscan time delayand the global cortical amyloid SUVR values, some biases may be present in thedata as a result of this non-negligible temporal delay. Lastly, while our studyrevealed statistically significant differences in qMT parameters on a*group*level, the significant overlap in these measuresbetweenAβ−andAβ+groups means that qMT biomarkers are currently not as sensitive as amyloid PETmeasures for binary signal detection tasks on an*individual*level.

### 4.2 Future directions

As the use of an unconstrained qMT model deviates significantly from the priorliterature, our study was designed to identify the MT parameters that mostclosely reflect the spatial distribution of fibrillar amyloid in neocorticalregions as compared with PET. Notably, our data suggest that$R_{1}^{\text{s}}$may be a promising parameter for this purpose. Our proof-of-concept study wasbased on a prototype qMT sequence originally designed for the study of whitematter. Future work will involve optimizing a sequence for the quantification ofthe Aβ burden in gray matter using the same procedure described inAssländer, Mao, et al. (2024).SupportingFigure S2shows that a pulse sequence optimized for further improved SNR in all six coreqMT parameters may be a promising avenue toward eliminating artifacts andimproving qMT’s overall sensitivity to the Aβ plaque burden.Additional improvements to the sequence are also needed to further reduce thescan time for routine clinical use. Further, the qMT maps exhibited artifactsparticularly affecting ROIs in the frontal and parietal lobes, which couldexplain their relative lack of significant effects compared with the temporallobe. One likely source of artifacts in the frontal and parietal lobes issubcutaneous fat. Future work will involve correcting for the chemicalshift-related artifacts to obviate the need for excluding ROIs in theanalysis.

The qMT sequence has an effective 1.24 mm isotropic resolution. Future work willexplore the potential advantages of this high resolution (as compared with PET)in the study of finer structures relevant to AD, including the hippocampus,cortex, and brainstem. For the latter, decreased neuromelanin and degenerationof the locus coeruleus in the pons have been associated with increased levels ofCSF Aβ (Betts et al., 2019) andvulnerability to the occurrence of neurofibrillary tangles (Braak et al., 2011) in early stage AD. As suggestedbyTrujillo et al. (2019), qMT couldpotentially be used to detect decreased neuromelanin in the locus coeruleus.

Intriguingly, our data in the subcortical gray matter show a significant decreasein both of the free pool’s relaxation rates$R_{1}^{\text{f}}$and$R_{2}^{\text{f}}$forAβ+participants, specifically in the hippocampus and thalamus (Fig. 5and SupportingTable S3). One possibleexplanation for these differences is inflammation (Leng & Edison, 2021), which is known to causedecreases in the relaxation rates (Stanisz etal., 2004). Iron is known to accumulate and colocalize with Aβin the hippocampal subiculum based on postmortem AD tissue samples (Madsen et al., 2020;Smith et al., 1997;Zeineh et al., 2015), and concentrations of the ferrous form, whichcauses oxidative stress, have been proposed to increase with microglial-driveninflammation (Tran et al., 2022), whichmay link Aβ pathology with subsequent neurodegeneration (Leng & Edison, 2021). However, our previousdata show that both$R_{1}^{\text{f}}$and$R_{2}^{\text{f}}$in the unconstrained qMT model are highly positively correlated with ironconcentration (Assländer, Mao, et al.,2024). Future studies could incorporate quantitativesusceptibility-weighted imaging (Ayton et al.,2017;Yedavalli et al., 2021)to verify possible hippocampal changes in iron content within the preclinical ADpopulation.

Unconstrained qMT can also be used to study injury in white matter areas (Makovac et al., 2018), where$m_{0}^{\text{s}}$was previously demonstrated to be closely associated with myelin content (Janve et al., 2013;Thiessen et al., 2013), but amyloid PET hassignificant off-target binding (Chapleau et al.,2022). Additionally, we hypothesize that cortical gray matterneurodegeneration in clinical AD would also be reflected by reduced$m_{0}^{\text{s}}$.Under the model of amyloid accumulation and progressive neurodegeneration astemporally displaced processes (Jack et al.,2010), this motivates further investigation into unconstrainedqMT’s sensitivity to patients with more advanced disease and specificassociations with the neurodegenerative axis of the A/T/N framework.Specifically, qMT’s potential sensitivity to both the “A”and “N” axes (Jack et al.,2018) might improve the specificity for and the monitoring ofprogression between preclinical, MCI, and dementia stages of AD. Along similarlines, more work is also needed to clarify whether qMT parameters are sensitiveto aggregates of tau proteins (like neurofibrillary tangles).

Mechanistic studies are needed to elucidate the specific pathophysiologicalprocesses underpinning the observed changes in$R_{\text{x}}$and$R_{1}^{\text{s}}$,including potential covariates such as blood–brain barrier breakdownleading to changes in perfusion (Tarasoff-Conwayet al., 2015), temperature differences (Birkl et al., 2013;Klegeris et al., 2006), or pH changes due to, for example,mitochondrial dysfunction (Louie et al.,2008;Wang et al., 2020).Additionally, a postmortem study of the relationship between$R_{\text{x}}$/$R_{1}^{\text{s}}$and neuritic plaque density would provide a stronger histopathological basis forour data. Lastly, our findings should be validated in preclinical (e.g., mouse)models of AD, as our unconstrained model differs from the existing literatureprimarily utilizing constrained qMT approaches (Bigot et al., 2014;Pérez-Torres et al., 2014;Praet et al., 2016).

While amyloid PET is a well-established technique with a simpler and more robustprocessing pipeline that offers much larger effect sizes, qMT—whichcombines high-resolution anatomical imaging and amyloid sensitivity in a singleexamination—could potentially be more amenable to screening, longitudinaland multicenter imaging studies by virtue of being implementable on existing MRIscanners. However, future work is still needed to establish the repeatabilityand reproducibility of the unconstrained qMT parameters, as well as streamliningthe image reconstruction and model fitting steps to reduce the technicalcomplexity. If these can be achieved, qMT imaging could potentially be usefulfor monitoring the longitudinal response to disease-modifying drugs in emergingclinical trials of antiamyloid immunotherapeutics, which are increasingly beingstudied in preclinical patient populations (Yadollahikhales & Rojas, 2023); e.g., in the AHEAD 3-45 study(NCT04468659).

## 5 Conclusion

Our study is the first to utilize an unconstrained qMT model to compare qMTparameters directly with an accepted measure of amyloid burden (amyloid PET) inpresymptomatic individuals on the Alzheimer’s disease spectrum. We show thatthe magnetization exchange rate and the semisolid spin pool’s longitudinalrelaxation rate are potential biomarkers of amyloid beta accumulation. While it doesnot achieve the sensitivity of amyloid PET, qMT is an augmentation to routinely usedconventional MRI that may enable the detection of amyloid accumulation withoutrequiring contrast agents or radiotracers. Future studies are needed to establish apotential role for qMT in monitoring disease progression and the response totherapy.

## Supplementary Material

Supplementary Material

## Data Availability

The qMT parameter maps, MPRAGE, and FBB PET SUV images for all participants areavailable athttps://osf.io/d6r4h/(doi:10.17605/OSF.IO/D6R4H). The source image reconstruction code used for theqMT data is available as a*Julia*package on Github athttps://github.com/JakobAsslaender/MRFingerprintingRecon.jl. For thepresented data, we used package v0.7.0 with Julia v1.10.0.*Julia*code to train the neural network used for qMT model fitting is also available onGithub athttps://github.com/andrewwmao/BiasReducedNetworks.
