# Supplementary material for: Sensitivity of unconstrained quantitative magnetization transfer MRI to amyloid burden in preclinical Alzheimer’s disease
Source: Imaging Neurosci (Camb). 2024 Nov 25;2:imag-2-00367. doi: 10.1162/imag_a_00367 (PMC12315764; doi:10.1162/imag_a_00367)
Supplement: Supplementary Material [file imag_a_00367-supp.pdf]

Supplementary Data for: Sensitivity of unconstrained quantitative magnetization transfer MRI to Amyloid burden in preclinical Alzheimer's disease

Andrew Mao,[a,b,c*] Sebastian Flassbeck,[a,b] Elisa Marchetto,[a,b], Arjun V. Masurkar,[d,e,f] Henry Rusinek,[a,b,d,g], Jakob Assländer[a,b]

[a]Bernard and Irene Schwartz Center for Biomedical Imaging, Department of Radiology,

New York University Grossman School of Medicine, New York, NY, USA

[b]Center for Advanced Imaging Innovation and Research (CAI^{2}R), Department of Radiology,

New York University Grossman School of Medicine, New York, NY, USA

[c]Vilcek Institute of Graduate Biomedical Sciences, New York University Grossman School of Medicine, New York, NY, USA

[d]Alzheimer's Disease Research Center, Center for Cognitive Neurology,

New York University Grossman School of Medicine, New York, NY, USA

[e]Department of Neurology, New York University Grossman School of Medicine, New York, NY, USA

[f]Department of Neuroscience and Physiology, New York University Grossman School of Medicine, New York, NY, USA

[g]Department of Psychiatry, New York University Grossman School of Medicine, New York, NY, USA

[*]Correspondence: andrew.mao@nyumc.org

September 15, 2024

References

Assländer, J., Mao, A., Marchetto, E., Beck, E. S., Rosa, F. L., Charlson, R. W., Shepherd, T. M., & Flassbeck, S. (2024). Unconstrained quantitative magnetization transfer imaging: disentangling

Cortical Lobe	FBB SUVR		m_0^s		R_x		R_1^s		Thickness	
	p	g	p	g	p	g	p	g	p	g
Frontal Ctx	$2{\cdot}10^{-8}$	3.49	0.200	0.49	0.104	−0.62	0.039	−0.78	0.851	0.10
Parietal Ctx	$2{\cdot}10^{-8}$	3.69	0.305	0.41	0.087	−0.60	0.019	−0.81	0.215	0.45
Temporal Ctx	$2{\cdot}10^{-8}$	2.32	0.124	0.63	0.022	−0.86	0.012	−0.94	1.00	0.01
Occipital Ctx	$2{\cdot}10^{-8}$	2.46	0.305	0.44	0.884	0.01	0.249	−0.48	0.232	0.28
Cort. Sig.	$2{\cdot}10^{-8}$	2.83	0.267	0.34	0.095	−0.76	0.087	−0.63	0.983	−0.03

Supporting Table S1: Summary of p (for the non-parametric Mann-Whitney U test) and Hedge's g values for the measures and ROIs shown in Figure 3. "Cort. Sig." refers to the "signature of AD-related cortical thinning" described in Dickerson et al. (2011). Insignificant p-values ($p > 0.05$) and their associated g's are shaded in grey.

Cortical Lobe	R_1^f		R_2^f		T_2^s	
	p	g	p	g	p	g
Frontal Ctx	0.692	0.23	0.573	−0.17	0.465	−0.33
Parietal Ctx	0.692	0.11	0.950	−0.18	0.723	−0.23
Temporal Ctx	0.215	0.37	0.285	0.31	0.662	0.12
Occipital Ctx	0.950	−0.05	0.465	−0.32	0.787	−0.27
AD-signature	0.787	0.11	0.851	0.04	0.185	−0.49

Supporting Table S2: Summary of p and g values for the remaining qMT parameters not shown in Figure 3. Insignificant p-values ($p > 0.05$) and their associated g's are shaded in grey.

ROI	FBB SUVR		m_0^s		R_1^f		R_2^f		Volume	
	p	g	p	g	p	g	p	g	p	g
Hippocampus	0.001	1.37	0.146	−0.28	0.005	−0.83	0.036	−0.87	0.107	−0.66
Amygdala	$1{\cdot}10^{-4}$	1.55	0.387	0.37	0.833	0.01	0.526	−0.26	0.076	−0.69
Thalamus	0.004	1.25	0.552	−0.26	0.009	−1.03	0.040	−0.78	0.863	−0.09
Caudate	$4{\cdot}10^{-8}$	2.34	0.064	−0.63	0.307	−0.48	0.158	−0.41	0.255	−0.43
Putamen	$9{\cdot}10^{-9}$	2.45	0.408	−0.39	0.064	−0.64	0.289	−0.43	0.924	−0.06
Pallidum	0.06	0.78	0.017	−0.95	0.008	−1.00	0.195	−0.60	0.346	−0.39
Global WM	$4{\cdot}10^{-7}$	2.52	0.107	−0.61	$7{\cdot}10^{-5}$	−1.54	0.010	−1.21	0.107	−0.72

Supporting Table S3: Summary of p and g values for the measures and ROIs shown in Figure 5. Insignificant p-values ($p > 0.05$) and their associated g's are shaded in grey.

ROI	R_x		R_1^s		T_2^s	
	p	g	p	g	p	g
Hippocampus	0.058	0.72	0.255	0.30	0.064	0.53
Amygdala	0.526	0.23	0.272	0.45	0.170	0.49
Thalamus	0.170	−0.64	0.255	0.46	0.010	1.0
Caudate	0.326	−0.40	1.00	0.07	0.985	0.11
Putamen	0.863	−0.18	0.833	−0.23	0.289	0.53
Pallidum	0.224	−0.49	0.716	0.04	0.346	0.32
Global WM	0.774	−0.04	0.687	−0.09	0.026	0.76

Supporting Table S4: Summary of p and g values for the remaining qMT parameters not shown in Figure 5. Insignificant p-values ($p > 0.05$) and their associated g's are shaded in grey.

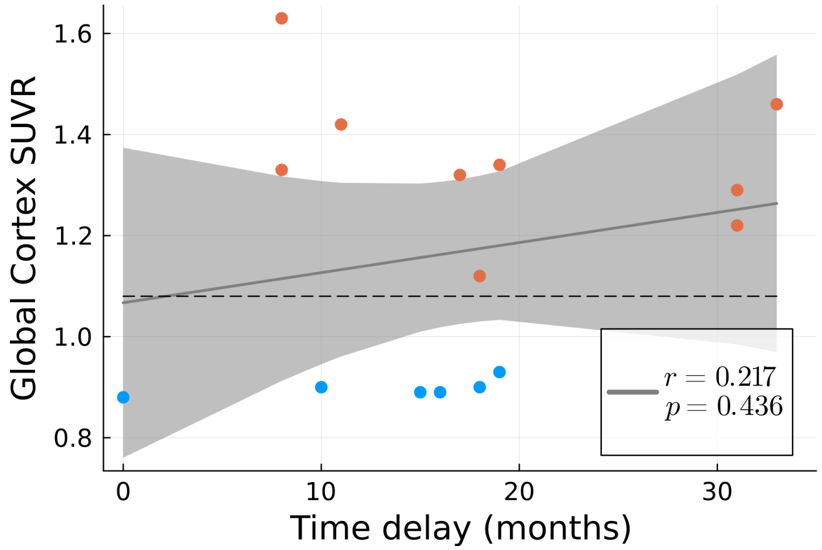

Supporting Figure S1: Scatter plot of the global cortical ^{18}F]Florbetaben (FBB) SUVR (computed as described in Section 2.8) as a function of the temporal delay between the FBB and qMT scans. The dashed line denotes the ^{18}F]Florbetaben (FBB) SUVR threshold of 1.08 used to discriminate Aβ− (blue) and Aβ+ (red) participants. There is no significant correlation between the inter-scan time delay and the global cortical amyloid burden.

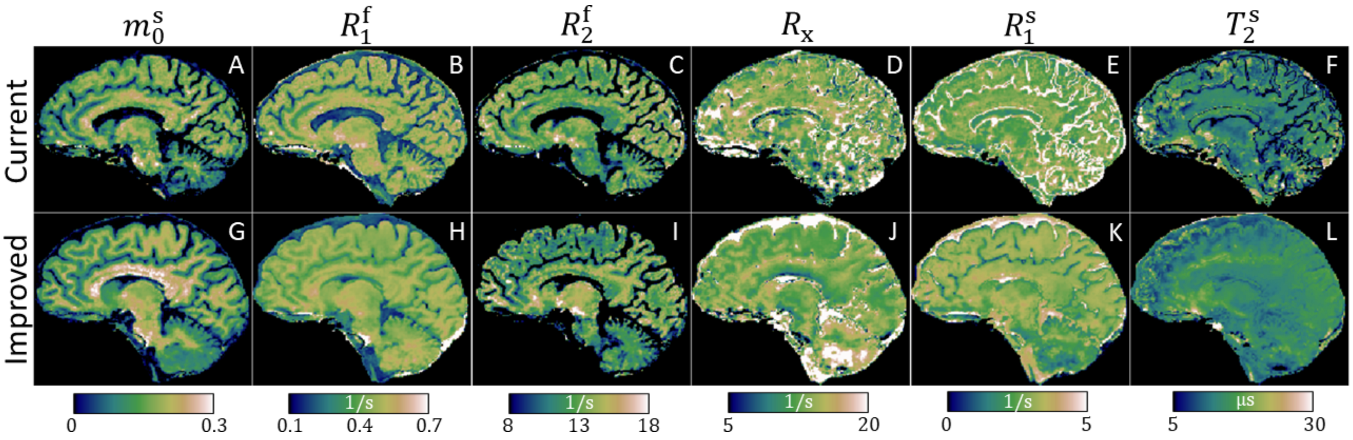

Supporting Figure S2: Comparison of qMT maps obtained using the RF pulse train described in Assländer et al. (2024) (A–F), which was used in this work, and an "improved" RF pulse train (G–L) that was optimized to further improve the overall SNR of all six qMT parameters. The six parameters are the semi-solid pool size m_0^s, the relaxation rates $R_{1,2}^{\mathrm{f,s}} = 1/T_{1,2}^{\mathrm{f,s}}$ (where the superscripts ^{f} and ^{s} denote the free and semi-solid spin pools, respectively), and the exchange rate R_{x}. The improved pulse sequence yields comparatively better image quality for R_{x} and R_1^{s} with fewer artifacts across the neocortex. This suggests that further optimization of the sequence for the quantification of R_{x} and R_1^{s} in cortical grey matter is a promising avenue for improving the sensitivity of qMT to Aβ plaque burden.

T1 of the free and semi-solid spin pools. *Imaging Neuroscience*, *2*, 1–16. https://doi.org/10.1162/imag_a_00177

Dickerson, B. C., Stoub, T. R., Shah, R. C., Sperling, R. A., Killiany, R. J., Albert, M. S., Hyman, B. T., Blacker, D., & Detoledo-Morrell, L. (2011). Alzheimer-signature MRI biomarker predicts AD dementia in cognitively normal adults. *Neurology*, *76*(16), 1395–1402. https://doi.org/10.1212/WNL.0b013e3182166e96
